# EEG revealed improved vigilance regulation after stress exposure under Nx4 – A randomized, placebo-controlled, double-blind, cross-over trial

**DOI:** 10.1016/j.ibneur.2021.09.002

**Published:** 2021-09-25

**Authors:** Tara Chand, Sarah Alizadeh, Hamidreza Jamalabadi, Luisa Herrmann, Marina Krylova, Galina Surova, Johan van der Meer, Gred Wagner, Veronika Engert, Martin Walter

**Affiliations:** a Department of Psychiatry and Psychotherapy, Jena University Hospital, Jena 07743, Germany; b QIMR Berghofer Medical Research Institute, Brisbane 4006, Australia; c Department of Psychiatry and Psychotherapy, University of Tübingen, Tübingen 72076, Germany; d Department of Psychiatry and Psychotherapy, Leipzig University Medical Center, Leipzig 04103, Germany; e Institute of Psychosocial Medicine, Psychotherapy and Psychooncology, Jena University Hospital, Jena 07443, Germany; f Department of Psychiatry and Psychotherapy, Philipps-Universität Marburg, Marburg, Germany; g Amsterdam UMC, Department of Radiology and Nuclear Medicine, 1105AZ, Amsterdam, The Netherlands

**Keywords:** Psychosocial stress, Vigilance, Resting-state, EEG, Neurexan

## Abstract

**Objectives:**

Vigilance is characterized by alertness and sustained attention. The hyper-vigilance states are indicators of stress experience in the resting brain. Neurexan (Nx4) has been shown to modulate the neuroendocrine stress response. Here, we hypothesized that the intake of Nx4 would alter brain vigilance states at rest.

**Method:**

In this post-hoc analysis of the NEURIM study, EEG recordings of three, 12 min resting-state conditions in 39 healthy male volunteers were examined in a randomized, placebo-controlled, double-blind, cross-over clinical trial. EEG was recorded at three resting-state sessions: at baseline (RS0), after single-dose treatment with Nx4 or placebo (RS1), and subsequently after a psychosocial stress task (RS2). During each resting-state session, each 2-s segment of the consecutive EEG epochs was classified into one of seven different brain states along a wake-sleep continuum using the VIGALL 2.1 algorithm.

**Results:**

In the post-stress resting-state, subjects exhibited a hyper-stable vigilance regulation characterized by an increase in the mean vigilance level and by more rigidity in the higher vigilance states for a longer period of time. Importantly, Nx4-treated participants exhibited significantly lower mean vigilance level compared to placebo-treated ones. Also, Nx4- compared to placebo-treated participants spent comparably less time in higher vigilance states and more time in lower vigilance states in the post-stress resting-state.

**Conclusion:**

Study participants showed a significantly lower mean vigilance level in the post-stress resting-state condition and tended to stay longer in lower vigilance states after treatment with Nx4. These findings support the known stress attenuation effect of Nx4.

## Graphical abstract

**Figure:**
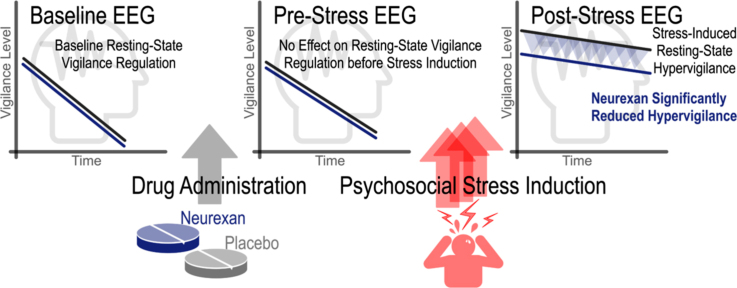

## 1 Introduction

Neurexan (Nx4) is a medicinal over-the-counter product in Germany which contains Avena sativa, Coffea arabica, Passiﬂora incarnata and Zincum isovalerianicum. It is approved by the German Federal Institute for Drugs and Medical Devices (German: Bundesinstitut für Arzneimittel und Medizinprodukte [BfArM]) for the treatment of sleep disorders and restlessness. Its effects have been demonstrated in several human (Doering et al., 2016, Herrmann et al., 2020, Hübner et al., 2009H, Waldschütz and Klein, 2008) and rodent studies (Dimpfel, et al., 2012). Thus, the intake of Nx4 for 28 days was shown to improve sleep quality by increasing sleep duration and reducing sleep latency as reported in patients’ sleep diaries over 14 days, and at the end of the study (Waldschütz and Klein, 2008). Taking Nx4 for two weeks led to a reduction in nervousness and restlessness in patients visiting German general practitioners (Hübner et al., 2009). A single dose of Nx4 (6 tablets over 2.5 h) reduced the acute neuroendocrine stress response measured in terms of stress induced changes in salivary cortisol and plasma adrenaline levels in healthy volunteers (Doering, Wegner et al. 2016). Studies of brain electrophysiology measured via electroencephalography (EEG) suggest a calming effect of Nx4. In rats, the drug increased the low-frequency delta and theta brainwaves (Dimpfel, et al., 2012). In humans, elevated frontotemporal beta2 power, a surrogate parameter of stress-induced anxiety, was dampened by the drug (Dimpfel, 2019). Further investigations are necessary to unravel the mode of action of Nx4 and better understand its beneficial effect on the acute stress response in humans.

Several behavioral states such as mood, stress, arousal, and vigilance, are determined by internal and external (environmental) stimuli such as motivationally salient or threatening stimuli, which are processed in the body by the autonomic nervous system and result in the release of specific hormones into the bloodstream. The immediate stress response in the body is characterized by the activation of both the autonomic nervous system (ANS) and the hypothalamic-pituitary-adrenal (HPA) axis, resulting in the release of catecholamines (adrenaline and noradrenaline) and corticosteroids. In the brain, particularly the noradrenaline producing locus coerueus (LC) and amygdala are playing a central role in the processing of salient information and mediating the effects of stress, e.g. due to threatening stimuli by triggering ANS response to stressors. The noradrenaline released by LC neurons subsequently modulates the stress or arousal response in frontal brain regions and modulates the activity of amygdala, inducing a rapid increase of attention and/or perception to/of a stimulus and changing the cortical arousal and vigilance level (Sara and Bouret, 2012, van Marle, et al., 2010; Veer, et al., 2011). In sum, the LC plays a central role in the induction and regulation of cortical arousal (Berridge, 2008 for comprehensive review) and induces a higher vigilance state under stress, a state referred to as tonic alertness by Oken et al. (2006). This higher vigilance state leads to a state of elevated sustained attention and alertness, and drives the stress response in associated brain regions including a faster processing of visual stimuli.

EEG is a reliable tool to investigate changes in vigilance states of the brain and can be used as a marker of cortical arousal. Brain vigilance states were shown to be altered by affective stimuli (Borchardt, et al., 2018), by stress and in the context of stress-related psychopathology (Klumpp and Amir, 2009, Silvers et al., 2017). For example, a significantly higher and prolonged vigilance level, and rigid, hyper-stable regulation of vigilance during rest were found in patients with obsessive-compulsive disorder (OCD) compared to healthy controls (Olbrich, et al. 2013). Investigating the effect of three attachment narratives on listeners’ cortical arousal, Borchardt et al. (2018) found that the high vigilance state caused by listening to any of the narratives decreased faster after an insecure-dismissing narrative than after insecure-preoccupied and secure narratives. Henckens et al. (2015) showed that neural vigilance processing varied depending on basal cortisol levels and stress-induced cortisol release following acute stress exposure. In detail, the authors demonstrated that acute stress affected the vigilance neurocircuitry, and that individual differences in neural stress responses were associated with differences in basal and stress-induced cortisol levels.

Staying aware of ones environment during stress exposure can be necessary for survival. Therefore, stress experience is characterized by a hyper-vigilance state, involving increased cortical arousal, tonic alertness, maintaining attention towards the external environment and fast responsiveness. A state of rest is contrarily characterized by a reduced vigilance state and cortical arousal (Olbrich, et al., 2013).

In the current study, vigilance states were estimated with an EEG-based algorithm (Vigilance Algorithm Leipzig, VIGALL 2.1) (Hegerl et al., 2012, Hegerl et al., 2017) which uses the cortical current density activity to classify different states of vigilance from high wakefulness to drowsiness until sleep onset during resting-state. We hypothesized that the participant group receiving Nx4 would show a lower resting-state vigilance following stress exposure than the placebo control group. To test this hypothesis, we investigated how the stress-induced vigilance regulation changed in resting-state EEGs before and after the intake of either Nx4 or placebo.

## 2 Methods

The current study is a post-hoc analysis from the NEURIM trial registered at ClinicalTrials.gov under the number NCT02602275 (date of registration: 28/10/2015), and approved by the ethics committee of the University of Magdeburg as well as the Competent Authority (Federal Institute for Drugs and Medical Devices). It complies with the ethical principles of the 1996 Declaration of Helsinki (Somerset West, Republic of South Africa), principles of the Good Clinical Practice (GCP) provided in the International Conference on Harmonisation (ICH) Harmonized Tripartite Guidelines for GCP 1996, and all applicable national laws and regulations. Prior to study participation, written informed consent was given by each participant after receiving detailed information on the study protocol from the principal investigator. The study took place at the Medical Faculty of the Otto-von-Guericke University of Magdeburg, Germany, from August 18th, 2015 to December 3rd, 2015. EEG and fMRI acquisitions were conducted at the university’s local imaging center. Participants who fulfilled the inclusion criteria were recruited from July to November 2015. All screenings and measurements were collected between August and December 2015.

### 2.1 Trial design

**Fig. 1:**
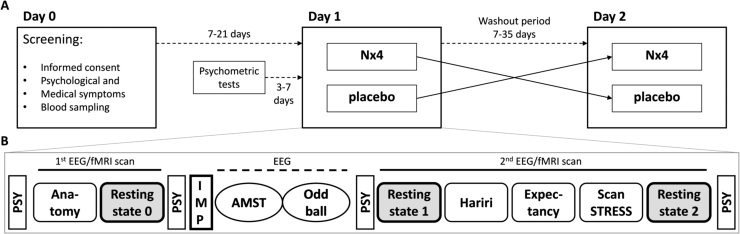
Design of the randomized, placebo-controlled, double-blind, two-period cross-over trial. A) Overall study design. On Day 0, participants gave informed consent, and psychological and medical symptoms were screened. IMP administration and EEG/fMRI measurements took place on each of both cross-over periods (Day 1 and Day 2). A minimum of 7 and a maximum of 21 days elapsed between Day 0 and Day 1. During this time and 3–7 days prior to Day 1, participants completed a battery of psychometric tests. The washout period between Day 1 and Day 2 amounted to 7–35 days. B) Study design of the cross-over EEG/fMRI sessions (as described in Herrmann et al., 2020). EEG/fMRI acquisition began with an anatomical scan followed by a baseline resting-state measurement. After intake of the IMP, the two EEG paradigms (AMST and Oddball) were performed. The second EEG/fMRI scan was comprised of three tasks, including stress induction via the ScanSTRESS paradigm, and two resting-state measurements before and after the tasks, respectively. Psychometric tests assessing anxiety and mood were completed before and after the first and second scans. PSY = Psychometric Tests; fMRI = functional Magnetic Resonance Imaging; IMP = Investigational Medicinal Product; EEG = Electroencephalography; AMST = Attention Modulation by Salience Task.

The NEURIM trial was conducted as a randomized, placebo-controlled, double-blind, two-period cross-over trial with an explorative design and confirmatory principles. Each participant attended three visits (Fig. 1A): On Day 0, medical and psychological symptoms and compatibility for MRI were screened. Psychometric testing of personality traits, life experiences and sensitivities were completed between 3 and 7 days prior to Day 1. Day 1 comprised the randomized distribution of participants to a respective treatment group, fMRI and EEG data acquisition. Considering the washout period, a cross-over EEG-fMRI session (Day 2) took place after 7–35 days from Day 1. Apart from the type of Investigational Medicinal Product (IMP), i.e., active ingredient versus placebo, study procedures were identical on both days (Day 1 and Day 2). Participants who received verum on Day 1 were given placebo on Day 2 and vice versa. Additional information of active ingredients in verum may be seen in Supplementary Table S1.

All resting state EEG measurements were acquired simultaneous with fMRI in two sessions (before and after the intake of IMP). All subjects were instructed to keep their eyes closed and do not engage in any specific tasks while trying not to fall asleep. A 12-minute baseline resting-state (RS0) was acquired before the intake of the IMP (Fig. 1B). Each participant got a single dose (three tablets) of either Nx4 or placebo 40–60 min before the second scan. During the interval between the first and the second resting-state scan (RS1 and RS2 respectively) EEG recordings were acquired while the participant performed two computerized tests, the Attention Modulation by Salience Task (AMST) (Dinica et al., 2016) and an auditory oddball task (Segalowitz and Barnes, 1993). The second scanning session comprised three tasks: 1) the Hariri task (Hariri et al., 2002), 2) the Expectancy task (Teckentrup et al., 2019), and 3) the ScanSTRESS task (a shortened version of the ScanSTRESS by Streit et al. 2014), as well as pre- (RS1) and post-task (RS2) eyes-closed resting-state scans of 12 min each. Psychometric tests measuring anxiety and mood were completed before and after the first and second scans. All fMRI measurements were realized with simultaneous EEG data acquisition.

To induce psychosocial stress, a shortened version of the ScanSTRESS task (Streit, Haddad et al. 2014), which is an adapted version of the Montreal Imaging Stress Task (Dedovic et al., 2005), was used. This paradigm consists of two runs of mental rotation and arithmetic calculation tasks, both of which include stress and control blocks. In the stress blocks, participants receive negative social evaluative feedback and perform difficult excercises under time pressure. Excercises in the control blocks are provided without negative feedback and are overall less demeaning than those of the stress condition.

### 2.2 Participants and inclusion criteria

**Fig. 2:**
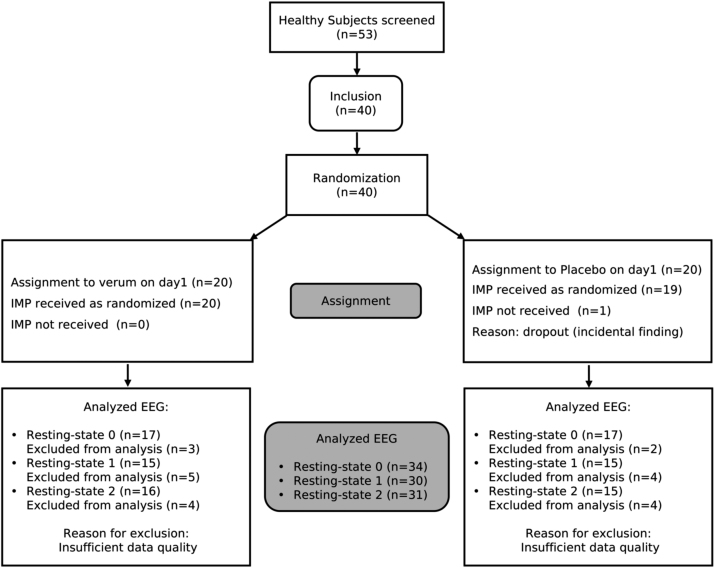
CONSORT flow diagram showing number of participants through each stage of the randomized cross-over trial.

Forty healthy male volunteers, between the ages of 31 and 59 (mean: 43.74 ± 9.81 years) were recruited. Volunteers were only included if they were physically healthy based on laboratory tests and a physical examination, MRI compatible, nonsmoking, and fluent in German. Subjects were screened for ongoing psychological diseases or episodes using the Structured Clinical Interview for DSM-IV (Wittchen et al., 1997). Chronic stress levels were determined using the screening version of the Trier Inventory for Chronic Stress (TICS; Schulz and Schlotz (1999)), and the Perceived Stress Scale (PSS; S. Cohen, Kamarck, and Mermelstein (1983)). Participants scoring within the range of mild to moderate stress (TICS Score ≥9 and ≤36, PSS > 9) were included. All others were excluded to ensure that participants were in principle susceptible to stress, and to avoid a ceiling effect of stress activity in cases of current severe or chronic stress experience. All subjects gave written informed consent before participation. Because during baseline MR measurements, one subject was excluded due to an incidental finding, 39 subjects were included in this study. Out of those 39 participants, only subjects with good-quality EEG data after preprocessing from both experimental days were included in the statistical analysis of each resting-state, resulting in 34, 30 and 31 subjects in baseline (RS0), pre-stress (RS1) and post-stress (RS2) resting-states, respectively.The flow of participants through each stage of the trial according to the CONSORT diagram is depicted in Fig. 2.

### 2.3 Blinding and randomization

Investigators and participants were blinded during the study period. Because verum and placebo contained lactose monohydrate and magnesium stearate, they were identical in taste, size, color, and labeling. As mentioned in the details of unblinding safety management, an investigator was given authority to break the treatment code in case of emergency only. There was no emergency unblinding reported during the study. A statistician not involved in the study generated the randomization codes within IDV Gauting, providing biometric services and strategic consulting. The randomization codes and the whole procedure were filed at a secure place by IDV Gauting until the study database was closed. Following the randomization list, a non-involved statistician also provided pack lists with medication numbers to Heel GmbH for manufacturing of the drug kits. A random allocation of participants to Nx4 or the placebo group was performed in a 1:1 randomization. A corresponding random code list was prepared, using the randomly permuted block scheme with a fixed block size. Participants were enrolled based on the randomization number recorded on the study medication. The next participant eligible for randomization was allocated to the lowest available randomization number at the site. A validated program RANCODE (Ver. 3.6) was used to prepare the sealed random code list and the sets of sealed envelopes in a standard working environment at idv Data Analysis and Study Planning, Krailling, Germany.

### 2.4 EEG data acquisition

In resting-state scans, EEG and fMRI data were acquired simultaneously with a Brain Products Easy Cap with 64-channels. All the subjects were instructed to keep their eyes closed and do not engage in any specific tasks while trying not to fall asleep. FCz and CPz electrodes were placed as reference and ground electrodes, respectively. One channel was placed on the subject’s back and was used for ECG detection. The data were recorded using BrainVision Recorder Professional V.1.20.0801 with a sampling rate of 5000 Hz. To increase the quality of the EEG recordings assessed in the simultaneous EEG-fMRI scans, the EEG cap was augmented with six carbon-wire loops (CWLs). Four CWLs were placed on the outer surface of the EEG cap at the left and right frontal and the left and right posterior locations. Two CWLs were attached to the cables connecting the EEG cap to the EEG amplifier (BrainAmpMR Plus). For further details on CWLs, see van der Meer et al. (2016).

### 2.5 EEG data preprocessing

To clean the resting-state EEG data from MRI gradient artifacts, we employed a state-of-the-art motion informed template subtraction method realized by the Bergen EEG-fMRI toolbox (Moosmann et al., 2009). For averaging and to obtain an MRI template waveform, 25 MRI artifacts in a sliding window were used (Allen et al., 2000). We used the realignment parameters from the fMRI analysis as interruption points, i.e. whenever the displacement vector reached a threshold value of 0.5 mm the window buffer was reset. After bandpass filtering of the artifact-corrected EEG data from 0.3 Hz to 200 Hz and downsampling to 1000 Hz, the helium pump and ballistocardiographic (BCG) artifacts were removed using the novel carbon-wire loop-based artifact correction technique (van der Meer, et al., 2016). This toolbox regresses out any EEG artifact which is correlated with the movements recorded by each of the six CWLs. Here we used Hann tapers with overlapping windows of 6 s length and a delay embedding of 21 ms as recommended by van der Meer, Pampel et al. (2016). Next, the data were segmented into 2 s epochs (i.e. equivalent to the repeation time (TR) of the BOLD resting-state scans) and the epochs containing muscle and head movements artifacts were removed from the data set in a semiautomatic process using custom MATLAB scripts. Channels that contained too many epochs with artifacts were also removed and interpolated using routines provided by EEGLAB (Delorme and Makeig, 2004). The final step of artifact rejection included the independent component analysis (ICA) decomposition of the EEG data and removing the components that reflected eye movements, heartbeat, continuous muscle activity and residuals of fMRI-artifacts.

### 2.6 Time course of vigilance regulation

To extract the time course of vigilance fluctuations and verify how the regulation of brain arousal changed after treatment with Nx4 compared to placebo, we performed an EEG-based vigilance classification algorithm on resting-state EEG data using the Vigilance Algorithm Leipzig (VIGALL 2.1; Hegerl, et al., 2017). The VIGALL 2.1 is a freely available Add-In for BrainVision Analyser 2.1 (Brain Products GmbH, Gilching, Germany). The algorithm requires several standardized pre-processing steps including filtering (0.5–70 Hz, 50 Hz Notch, 48 dB Slope), downsampling (100 Hz) and manual marking the indicators of sleep onset (sleep spindles and K-complexes), as these markers are used by VIGALL to classify C-stages (for detailed description please see the manual: Hegerl et al., 2017). The VIGALL classification is based on the distribution of cortical current density activity over four distinct regions of interest (occipital, parietal, temporal, frontal). An estimate of the cortical current density is separately computed for the delta/theta and the alpha frequency range using the LORETA method (Pascual-Marqui, et al., 1994). The delta/theta band was set between 3 and 7 Hz. Each subject’s individual alpha center frequency was automatically detected, and the alpha band was defined as ±2 Hz around the individual alpha center frequency. Vigilance states in 355 consecutive EEG epochs (each had a duration of 2 s) were estimated in each resting-state (RS0/RS1/RS2) and condition (verum/placebo). This was the maximum length of recording available for each subject. Each segment was automatically classified into one of seven different brain states along a wake-sleep continuum (alertness: 0, A1, A2, A3; drowsiness: B1, B2/3; and sleep onset: C). Stage 0 is characterized by low-amplitude non-alpha EEG. In stages A1-A3 dominant alpha activity is prevalent in occipital (A1), central-frontal (A2) and frontal (A3) areas. Stage B1 reflects low-amplitude non-alpha EEG with slow eye movements. In stage B2/3 dominant delta and theta powers are observed. Sleep onset stage C is characterized by the appearance of sleep spindles and/or K-complexes. Because electrooculography was not available, differences of the signals between two frontal electrodes F7 and F8 were used to estimate horizontal eye movements. To compare the time course of vigilance fluctuations, we assigned a vigilance score ranging from 1 to 7 to each EEG-vigilance state (1 represents the lowest (stage C) and 7 the highest (stage 0) vigilance state), and averaged the vigilance scores within non-overlapping 2-min intervals, resulting in 6 time points (equivalent to 12 min) for each vigilance curve.

### 2.7 Statistical analysis

To examine the drug effect on the time course of vigilance fluctuations, a repeated-measures ANOVA with two main factors of treatment (verum vs. placebo) and time (6 different time points in the vigilance curve) as within-subject factors and treatment-sequence (placebo-verum or verum-placebo) as a between-subject factor was performed in each resting-state. The Greenhouse-Geisser correction was applied if the sphericity assumption was violated. To examine the drug effect on the slope and offset of the vigilance curve, a line was fitted to the vigilance curve of each subject using linear regression analysis. The resulting slopes/offsets were compared across subjects using paired T-tests. Because the data was not normally distributed, percentages of vigilance states under Nx4 and placebo were compared using the two-sided Wilcoxon signed-ranked test. Only those subjects that had good-quality EEG data after preprocessing from both experimental days were included in the statistical analysis of each resting-state, resulting in 34, 30 and 31 subjects in RS0, RS1 and RS2, respectively.

## 3 Results

### 3.1 Temporal dynamics of vigilance levels before and after stress exposure

**Fig. 3:**
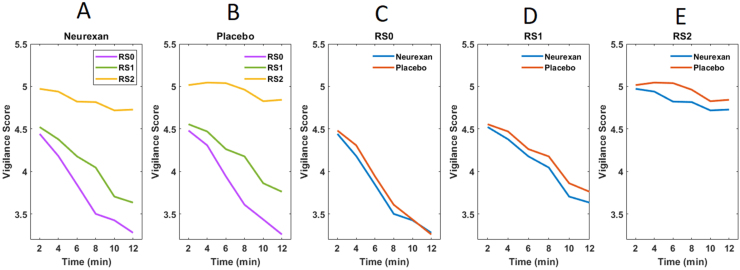
Time course of EEG-vigilance fluctuations under Neurexan (A) and placebo (B) in three different resting-state recordings. For the sake of comparison, vigilance curves under Neurexan and placebo are shown in each resting-state recording separately (C-E). Each of the six time points of the curve corresponds to the average vigilance score in a 2-min window.

The time course of EEG vigilance fluctuations averaged over subjects in the resting-state recordings during baseline (RS0), before, and after exposure to stress (RS1 and RS2 respectively) is shown for verum and placebo conditions separately in Fig. 3. During RS0 and RS1, subjects exhibit progressive decline to lower vigilance levels over the time of scanning (Fig. 3C and D). In contrast, subjects continuously remain in high arousal states (hyper-stable arousal regulation) during RS2 after the stress task (Fig. 3E).

To quantify the general effect of stress on the slope and offset of the vigilance curve, we performed paired *t*-tests to compare data before and after stress from the placebo session only (RS2 vs. RS1). We found that acute stress induction significantly decreased the steepness [t(29) = 3.82, p = 0.0005] and increased the offset [t (29) = 2.23, p = 0.032] of the vigilance curve.

### 3.2 Changes in slope and offset of vigilance curves after the treatment with Nx4

Repeated-measures ANOVA with within-subject main factors of treatment (verum vs. placebo) and time (6 different time points of the vigilance curve) in each resting-state showed significant main effects of treatment (F(1,29) = 6.53, p = 0.016) and time (F (2.52,29) = 3.26, p = 0.033, after Greenhouse-Geisser correction) on the vigilance score only in post-stress resting-state (RS2). No significant carry-over effect (treatment × treatment-sequence interaction: F (1) = 0.318, p = 0.58) or treatment × time interaction (F (1,3.43) = 0.352, p = 0.814) was found. Wilcoxon test for paired samples confirmed that Nx4 significantly reduced the mean/overall vigilance level of subjects only in RS2 (Z = −2.645, p = 0.008). No significant result was found in RS1 or RS0. Repeated-measures ANOVA with main factors of treatment (verum vs. placebo) and resting-state (RS1 vs. RS2) showed no significant treatment or treatment x resting-state interaction.

Testing for the effect of Nx4 on the slope and offset of the vigilance curves using paired *t*-tests showed that Nx4 reduced the offset of the vigilance curve after stress (trend level: t(30) = −1.89, p = 0.068), but had no significant effect on the slope of the curve (t(30) = 0.315, p = 0.755). No significant drug effect was found on the slope or offset of the vigilance curve in RS1 or RS0.

### 3.3 Prevalence of different vigilance stages

Comparing the percentage of EEG-vigilance stages 0, A1, A2, A3, B1 and B2/3 in the verum vs. placebo conditions using Wilcoxon signed-rank test revealed significant differences between Nx4 and placebo only for B2/3 in RS2. The percentage of EEG segments with the low vigilance stage B2/3 was significantly higher under Nx4 compared to placebo (mean in %±SD: Nx4 = 11.02 ± 21.17, placebo = 3.76 ± 8.70; p = 0.006). No significant difference was found in RS1 or RS0.

### 3.4 Behavioral correlations with stress-induced changes in mean vigilance

**Fig. 4:**
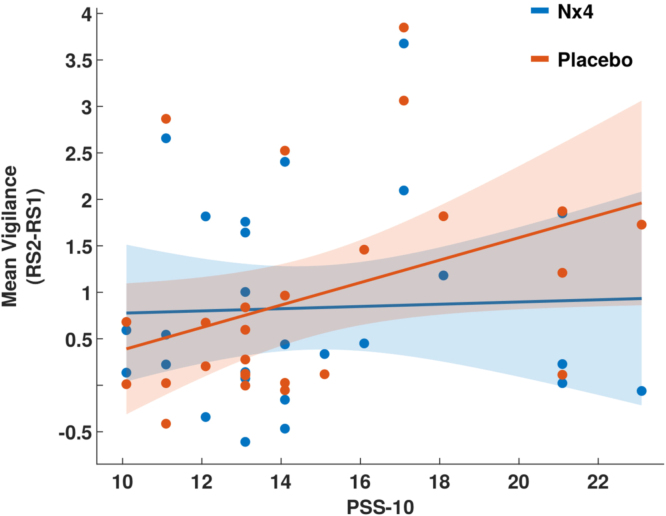
The relationship between the stress induced changes in mean vigilance with the perceived stress scale measured by PSS-10 in placebo group (red) and Nx4 group (blue). The correlation is significant only in placebo condition (R = 0.46, p = 0.02).

We further analyzed the associations of the stress-induced changes in mean vigilance with perceived stress scale measures like TICS and PSS-10. To do this, we compared the stress-induced changes in vigilance (RS2-RS1) with the PSS-10 and TICS in the placebo and verum groups separately. No significant correlation between TICS and the delta mean vigilance (RS2-RS1) in the verum or placebo groups was found. However, we found that the stress-induced changes in mean vigilance is positively correlated with PSS-10 scores in the placebo group (R = 0.46, p = 0.02, see Fig. 4). This means that subjects with higher perceived stress scale based on PSS-10 have more increase of the mean vigilance after stress induction if they are treated with placebo. There is no correlation when subjects are treated with Nx4 (See below Fig. 4).

## 4 Discussion

EEG signals act as a marker of arousal regulation in the brain. In the current study, we showed that placebo-treated control subjects exhibited a hyper-vigilance state characterized by an increase in the mean vigilance level and by more rigidity in the higher vigilance states for a longer period of time after acute psychosocial stress experience. In other words, during the resting-state following acute stress exposure (RS2), participants were more alert and tense, and showed a hyper-stable vigilance regulation in the placebo condition. This state was quantified in terms of an increase in the offset and a decrease in the steepness of the vigilance curve in post– compared to pre-stress conditions. Subjects treated with the over-the-counter product Neurexan (Nx4) as compared to placebo had significantly lower mean vigilance levels, spent significantly more time in the lower vigilance state B2/3 and exhibited a decrease in the offset of their vigilance regulation after stress. These findings show that Nx4 reduces the effects of stress on vigilance regulation by decreasing the offset (but not the slope) of the vigilance curve, thus indicating a stress attenuating effect of Nx4.

In the pre-stress resting-state measurements (RS0 and RS1), subjects showed a steeper decline in the vigilance state as compared to after the acute stress task (RS2). Further, we found a significant decrease in the steepness and increase in the offset after the stress task (RS2). This shows a hyper-stable arousal regulation after the stress task and represents a significant stress effect on arousal regulation.

Considering the treatment effect, a significant decrease in the offset but not the steepness of the vigilance curve in post-stress resting-state (RS2) under Nx4 as compared to placebo suggests that Nx4 does not change the dynamics of the vigilance regulation over time (slope), but rather decreases the mean vigilance level by attenuating the stress response and calming the subject. Interestingly, no significant drug effect was observed in RS1. In the pre-stress condition, both placebo and drug group showed a typical vigilance decline in the scanner (Tagliazucchi & Laufs, 2014). Absence of a significant difference between Nx4 and placebo in RS1 indicates that reduction in the mean vigilance level after stress under Nx4 was not due to a sleep-inducing effect but rather a stress-attenuating effect of Nx4.

The change in stress response under Nx4 may relate to its effect on amygdala activity. Activation of the amygdala and an increase in the vigilance level help to quickly react to threatening situations (Klumpp and Amir, 2009). In a similar line of argument, previous studies showed increased amygdala activity in stress-related disorders (Drevets, 2003, Etkin and Wager, 2007). Interestingly, recent investigations in the current participant sample (Herrmann et al., 2020) showed lower activity in the centromedial amygdala (CeMA) response to negative stimuli (emotional face matching, Hariri task) in the Nx4 compared to the placebo group. The CeMA has efferent projections to cholinergic neurons (Davis and Whalen, 2001, Fadok et al., 2018). Via these projections, amygdala activation indirectly affects the whole cortex thus playing an important role in modulating general arousal (Davis and Whalen, 2001). Furthermore, the CeMA-thalamus coupling helps to maintain sustained attention on potential threats and modulates the vigilance level (Torrisi et al., 2018, Cain et al., 2002, Fadok et al., 2018). Taken together, our results suggest that the Nx4-induced alteration of the vigilance level might be mediated by the amygdala which is critically involved in the stress response by altering the vigilance level.

Schulz et al. (1998) previously tested Valeriana officinalis and Passiflora incarnata for their sleep-inducing effects and the capacity to compensate the stimulating effect of caffeine using self-report ratings and quantitative EEG (qEEG) in a placebo-controlled study with female subjects. They found Valeriana officinalis to show a significant effect on the subjective ratings of sedative effects. However, qEEG could not reliably predict sedation. Nevertheless, the drugs affected the qEEG signals. Valeriana officinalis increased delta, theta and slow alpha bands, whereas Passiflora incarnata only increased theta power. qEEG was also used by Schellenberg et al. (1993) who found that a phytopharmacological drug containing Radicis valerianae and Herbae passiflorae incarnatae increased theta- and alpha-power after two weeks of treatment in psychosomatic and affective patients. Similar to these findings, we found that the Nx4 group spent more time in the low vigilance state B2/3 in the post-stress resting-state, which represents dominant delta and theta power. Importantly, spending more time in lower vigilance states under Nx4 does not necessarily mean more drowsiness or loss of attention in the Nx4 group. In contrast, our results show no significant difference in arousal regulation (i.e., no change in the offset or slope of the vigilance curve) after Nx4 compared to placebo intake in the pre-stress resting-state condition (RS1). In fact, the modulating effect of Nx4 on vigilance regulation is evident only in the post-stress resting-state condition (RS2). Additionally, the stress-induced changes in vigilance show a positive correlation with PSS-10 only in the placebo group, suggesting that participants with higher perceived stress scale based on PSS-10 more increases in vigilance level after stress induction if they are treated with placebo. Interestingly, there is no correlation if participants are treated with Nx4 which could be due to stress-reducing effect of Nx4. Taken together, our results suggest that the stress-reducing effect of Nx4 may be due to its calming influence without deteriorating attention and concentration.

Several limitations of the present study should me mentioned. As no electrooculography was available during the recording, horizontal eye movements were estimated to consider this correction factor. Also, the EEG was acquired inside the MR scanner, which made it necessary to introduce additional corrections to the data. Lastly, since our sample is comprised only of healthy, middle-aged and mildly to moderately stressed male subjects, the generalizability of the current results to diseased patients should be considered with caution.

## 5 Conclusion

We found that in the post-stress resting-state, subjects exhibit a hyper-vigilance state (quantified as a rigid, hyper-stable vigilance curve), and their vigilance regulation does not show the decline typically observed during resting-state. However, in the resting-state following psychosocial stress, subjects under Nx4 exhibited significantly lower mean vigilance levels, spent significantly more time in lower vigilance states and exhibited a decrease in the offset of their vigilance regulation when compared to a matched placebo group. These findings indicate that Nx4 brings the post-stress vigilance regulation to a normal decline due to its stress-attenuating effects. However, the effect of Nx4 on chronically stressed individuals has yet to be investigated. Furthermore, in addition to the effect of Nx4, psychotherapy and behavioral modification might be very important to treat/prevent increased perceived psychosocial stress. However, this is out of the scope of this study and may be investigated in future studies.

## Declaration of competing interests

M.W. received institutional research support from Heel paid to his institution for this study, and from BrainWaveBank, H. Lundbeck A/S and LivaNova Belgium N.V., LivaNova PLC outside the submitted work. The University of Tübingen received institutional fees for advisory services by Prof. Walter from Heel GmbH, Servier Deutschland GmbH, Bayer AG and Janssen-Cilag GmbH. The University of Tübingen received financial support for conference attendance of T.C., S.A., L.H., M.K. and H.J. from Heel for presenting data of this study not reported in this article. L.H., J.V.M., T.C., S.A., H.J., and M.K. were part of M.W. team for this study, and declare no other conflict of interest outside the submitted work. All investigators followed the institutional guidelines for COI management in full compliance with the regulations of the Otto v. Guericke University, Magdeburg.
