# Supplementary material for: EEG revealed improved vigilance regulation after stress exposure under Nx4 – A randomized, placebo-controlled, double-blind, cross-over trial
Source: IBRO Neurosci Rep. 2021 Sep 25;11:175–82. doi: 10.1016/j.ibneur.2021.09.002 (PMC8545679; doi:10.1016/j.ibneur.2021.09.002)
Supplement: Supplementary Table S1 — Supplementary material [file mmc1.docx]

**Supplementary Table S1.** Qualitative and quantitative composition of Neurexan^®^.

| **Active substance (homeopathic denomination)** | **Used plant part / Starting material GHP method** | **Potency (Calculated potency corresponding to final dilution)** | **Mass per 1 tablet (mg)** | **Amount per daily standard dose (3 tablets)** | **Amount per maximum daily dose (12 tablets)** |
| --- | --- | --- | --- | --- | --- |
| Avena sativa  Avena sativa L. | Fresh, aerial parts harvested during flowering season GHP method 1a  dry residue ≥ 2 %;  ǿ = ½ part pressed juice | 2 | 0.6 | 1.8 mg D2  = 180 µg D1  = 36 µg mother tincture (contains  18 µg expressed juice) | 7.2 mg D2  = 720 µg D1  = 144 µg mother tincture (contains  72 µg expressed juice) |
| Coffea arabica  (Coffea)  Coffea arabica L. | Ripe, dried, unrosted seeds deprived from the exocarp  GHP method 4a; ǿ ≥ 0.1% caffeine | 12 | 0.6 | 1.8 mg D12  = 1.8 x 10^-11^ mg D1  = mother tincture  with at least 1.8 x 10^-14^ mg alkaloids | 7.2 mg D12  = 7.2 x 10^-11^ mg D1  = mother tincture  with at least 7.2 x 10^-14^ mg alkaloids |
| Passiflora incarnata  Passiflora incarnata L. | Fresh aerial parts GHP method 3a  dry residue ≥ 1.6 % | 2 | 0.6 | 1.8 mg D2  = 180 µg D1  = 54 µg mother tincture | 7.2 mg D2  = 720 µg D1  = 216 µg mother tincture |
| Zincum  isovalerianicum  (Zincum  valerianicum)  Zinc oxide +  isovalerianic acid  Zn(C5H9O2)2 x  2H2O | Zinc isovalerianate (in German: Baldriansaures Zink) GHP method 5a  D2 = 0.93 – 1.08 %  substance triturations HAB 6 D1 = 9.3 –  10.8 % substance | 4 | 0.6 | 1.8 mg D4  = 18 µg D2  with ca. 0.18 µg substance | 7.2 mg D4  = 72 µg D2  with ca. 0.72 µg substance |

Abbreviations: D = decimal potency, GHP = German Homeopathic Pharmacopoeia, Homöopathisches Arzneibuch (HAB)
